# Improvement of Rice Blast Resistance in TGMS Line HD9802S through Optimized Anther Culture and Molecular Marker-Assisted Selection

**DOI:** 10.3390/ijms241914446

**Published:** 2023-09-22

**Authors:** Pingli Chen, Guanjun Gao, Guangming Lou, Jie Hu, Yufu Wang, Rongjia Liu, Da Zhao, Qing Liu, Bingrui Sun, Xingxue Mao, Liqun Jiang, Jing Zhang, Shuwei Lv, Hang Yu, Wenfeng Chen, Zhilan Fan, Chen Li, Yuqing He

**Affiliations:** 1 National Key Laboratory of Crop Genetic Improvement, National Center of Plant Gene Research, Hubei Hongshan Laboratory, Huazhong Agricultural University, Wuhan 430070, China; 2 Rice Research Institute, Guangdong Academy of Agricultural Sciences, Guangdong Key Laboratory of New Technology in Rice Breeding, Guangzhou 510640, China; 3 Guangdong Key Laboratory of Genetics and Breeding of High Quality Rice in Southern China (Co-Construction by Ministry and Province), Ministry of Agriculture and Rural Affairs, Guangzhou 510640, China

**Keywords:** rice blast resistance, thermosensitive genic male sterile line, anther culture, marker-assisted selection

## Abstract

Rice blast caused by *Magnaporthe oryzae* is one of the most serious rice diseases worldwide. The early *indica* rice thermosensitive genic male sterile (TGMS) line HD9802S has the characteristics of stable fertility, reproducibility, a high outcrossing rate, excellent rice quality, and strong combining ability. However, this line exhibits poor blast resistance and is highly susceptible to leaf and neck blasts. In this study, backcross introduction, molecular marker-assisted selection, gene chipping, anther culture, and resistance identification in the field were used to introduce the broad-spectrum blast-resistance gene *R6* into HD9802S to improve its rice blast resistance. Six induction media were prepared by varying the content of each component in the culture medium. Murashige and Skoog’s medium with 3 mg/L 2,4-dichlorophenoxyacetic acid, 2 mg/L 1-naphthaleneacetic acid, and 1 mg/L kinetin and N6 medium with 800 mg/L casein hydrolysate, 600 mg/L proline, and 500 mg/L glutamine could improve the callus induction rate and have a higher green seedling rate and a lower white seedling rate. Compared to HD9802S, two doubled haploid lines containing *R6* with stable fertility showed significantly enhanced resistance to rice blast and no significant difference in spikelet number per panicle, 1000-grain weight, or grain shape. Our findings highlight a rapid and effective method for improving rice blast resistance in TGMS lines.

## 1. Introduction

More than 50% of the global population relies on rice (*Oryza sativa* L.) as a staple food. Rice blast is a fungal disease caused by *Magnaporthe oryzae*, which is widely distributed worldwide [1]. Rice blast can harm rice throughout its growth period and can be divided into leaf, neck, and grain blasts. The harm caused by neck blast is greater than that caused by leaf blast and other types of blast, and in severe cases, it can lead to a complete lack of harvest [2]. Every year, 10–30% of the world’s rice yield is lost because of the rice blast [3]. With rapid changes in the virulence characteristics of blast fungi, the resistance of existing blast-resistant varieties is easily overcome, and resistance is lost. Therefore, it is particularly important to breed varieties with long-term resistance [4].

Research has shown that searching for broad-spectrum and long-lasting resistance genes and selecting and applying resistant varieties are the most economical and effective methods for controlling rice blast [5]. To date, more than 100 main blast-resistance genes have been reported, among which a large number have been cloned [6,7,8], such as *Pi1*, *Pi2*, *Pi9*, *Piz-t*, *Pigm*, *Pikm*, *Pikh*, *Pish*, *Pita*, *Pi21*, *Pit*, *Pi25*, *Pi35*, *Pi36*, *Pi37*, *Pi50*, *Pi56*, *Pi64*, *Pi-d2*, and *Pi-d3*. *Pi1* and *Pi2* are the two major blast-resistance genes, both of which are dominant broad-spectrum resistance genes [9,10]. Both originate from *indica* rice and are located on chromosomes 11 and 6, respectively [10]. 75 rice blast strains were collected from different regions for inoculation, and it was found that the resistance spectrum of lines carrying *Pi2* reached 92.45%. *Pi2*, *Pi9*, *Pi50*, *Pigm*, *Piz*, and *Piz-t* are multiple alleles at the same locus [11,12], with Erbazhan carrying *Pi50* [13], while *Pi9* comes from small-grain wild rice [14]. Genes such as *Pi9* [15], *Pigm* [5], *Pikh* [16], and *Bsr-d1* [17] not only have strong resistance but also exhibit broad-spectrum resistance and persistence. Studies such as those cited above provide important support for the use of disease-resistance genes for genetic improvement in rice.

Rice genome sequencing and the development and application of molecular markers are efficient technologies that can replace traditional breeding, facilitating the utilization of specific gene resources [12,18]. Traditional breeding requires the creation of specific environments for identification and screening. Marker-assisted selection (MAS) is an effective method for transferring favorable genes for genetic improvement because of its ability to quickly and accurately select target genes that are not limited by the rice growth period [11,12,18,19]. Their application is particularly important for breeding resistant rice varieties. MAS can be applied quickly and accurately for the genetic improvement of rice blast-resistance genes that have been finely mapped and cloned. The broad-spectrum blast-resistance gene *Pi9*(t) from the donor parent P2 was introduced into the hybrid rice restorer line Luhui17, and backcrossing and MAS were used to introduce *Pi2* into Zhenshan 97 B [20]. MAS was used to develop a sterile male line, Jin 23A, carrying both *Pi1* and *Pi2* [21]. The disease resistance of four male-sterile lines, Y58S, Guangzhan63s, C815S, and HD9802S, was improved by introducing nine cloned genes, *Pi37*, *Pit*, *Pid3*, *Pigm*, *Pi36*, *Pi5*, *Pi54*, *Pikm*, and *Pb1*, with broad-spectrum blast resistance [11]. Three broad-spectrum blast-resistance genes, *Pi9*, *Pi5*, and *Pi54,* were integrated into Wuhan1S using MAS, conventional hybridization, and high-intensity stress screening methods [22].

There are many techniques in rice breeding, including anther culture (AC), which is widely applied to many crops, such as rice, wheat, and barley [23,24,25]. Many high-quality *japonica* and *indica* rice varieties obtained through AC, as well as hybrid rice, have significantly contributed to the increase in agricultural yield. One of the main advantages of AC is its ability to quickly aggregate several genes, thereby accelerating the breeding process and shortening breeding time [23,25]. However, some bottlenecks limit the widespread utilization of in vitro anther culture in rice breeding, especially in *indica* genotypes [25,26,27]. There are many parameters of culture that affect the efficiency of AC, such as carbon source, induction medium (IM), temperature, and growth regulator [25,28,29,30]. There have been many reports on the different IM and culture conditions and other related aspects of AC [25,26,27]. However, to our knowledge, there are few reports on the improvement of thermosensitive genic male sterility (TGMS) in *indica* rice through the use of MAS and AC [31,32].

The TGMS line HD9802S was bred by Hubei University [33,34]. Male sterile plants were found in the F_2_ segregated populations of Huda51 and Hongfuzao, which were bred into HD9802S by genealogical low temperature selection and artificial climate box identification. Through identification of rice blast in the field, it was found that the rice blast of HD9802S was at level 5 and the variety was highly susceptible to rice blast [11,33]. In the early stages of this study, under natural field-induced conditions, the blast resistance gene *R6* was identified using a recombinant inbred line constructed from the high-rice-blast-resistant cultivar 194-3 and the rice-blast-susceptible cultivar Zhenshan97B [32,35]. *R6* is located on chromosome 6 and encodes the entire growth period of the rice-blast-resistance gene [35]. In the present study, the *R6* gene was introduced into HD9802S through conventional hybridization and then backcrossed to construct BC_3_F_1_. By combining MAS, gene chips, AC, and field identification of rice blast resistance to select target genes, HD9802S was further improved to enhance rice blast resistance.

## 2. Results

### 2.1. Introgression of Blast-Resistance Gene R6 into the Background of HD9802S

**Figure 1:**
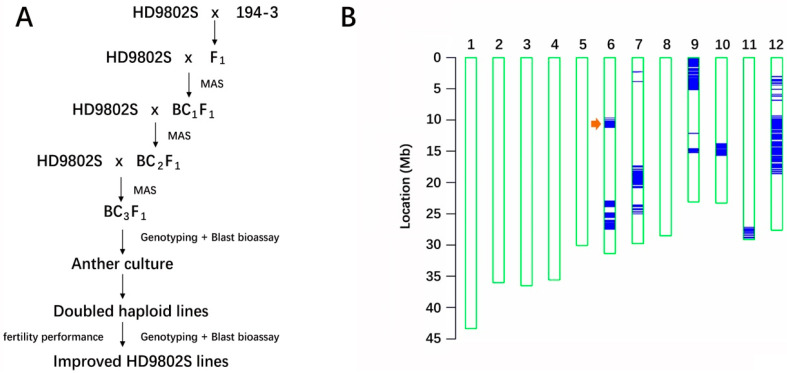
Strategy to develop improved populations and resistance identification. (**A**) Scheme of marker-assisted backcross breeding for improved TGMS line stacking *R6* coupled with anther culture used in the current study MAS: marker assistance selection. (**B**) Haplotype map of genetic background profiling using the RICE6K array The arrow indicates the position of *R6* on chromosome 6. The blue lines indicate the SNP loci with heterozygote genotypes where genomic fragments of the donor parent 194-3 were introgressed.

Through three backcrossing processes combined with MAS, the rice-blast-resistance gene *R6* gradually infiltrated from the donor parent 194-3 into the recurrent parent HD9802S, a TGMS line with high rice-blast sensitivity, which was improved in this study. The parent material 194-3 has been verified as a durable, high-blast-resistance material in Yuan’an. The *R6* gene originated from 194-3 and has been a persistent high rice-blast-resistance gene identified in our laboratory for many years [35]. *R6* was also a target gene for improving HD9802S blast resistance using MAS. The parent 194-3 was hybridized with the recipient parent HD9802S, resulting in F_1_ backcrossing with HD9802S to obtain BC_1_F_1_ and continued backcrossing with HD9802S twice to obtain BC_3_F_1_ (Figure 1A). In each generation of the backcross population, the molecular markers L11-1 and I6 on the two flanking regions of *R6* were used for MAS to identify positive individuals. Further backcrossing was conducted using HD9802S (Figure 1A,B), and 35 plants containing *R6* with heterozygous molecular markers on both sides were screened. Markers are used to select for progeny with target genes and closely linked flanking markers to produce chromosomes containing target alleles that are surrounded by minimal DNA from the donor parent (minimizing linkage drag), referred to as “negative selection” or “recombination selection” [36]. Markers distributed across all 12 rice chromosomes can be selected to restore the recurrent parent genome, which is known as “background selection” [36]. We used the whole-genome single-nucleotide polymorphism (SNP) breeding chip RICE6K [37] to analyze the genetic negativity and background of a single plant randomly selected from BC_3_F_1_ (Figure 1B). The chip contained 1310 polymorphic SNP markers between 194-3 and HD9802S. After three backcrosses, the genetic background recovery of the recurrent parent in a single plant from BC_3_F_1_ was about 90.93%, measured by the percentage of the polymorphic marker ratios. There were large donor chromosome fragments near *R6* on chromosome 6 (Figure 1B), which were the result of MAS foreground selection of the target gene in each generation. The two flanking molecular markers were heterozygous, and BC_3_F_1_ plants with similar genetic backgrounds, agronomic traits, and high resistance to rice blast were selected for AC.

### 2.2. Anther Culture

From the BC_3_F_1_ plants containing *R6*, those with a concentrated plant type and more tillering (sterile or semi-sterile) were screened, and the main stems during the late uninucleate stage were selected for AC. Six IMs were used for AC, with 26,600 anthers grafted (Table 1). Among the six IMs, only IM5 and IM6 were N6 media, while the others were Murashige and Skoog’s (MS) media. The proportions of 2,4-dichlorophenoxyacetic acid (2,4-D), 1-naphthaleneacetic acid (NAA), kinetin (KT), casein hydrolysate (CH), proline (Pro), and glutamine (Glu) in the six IMs were slightly adjusted. After 25 d of dark cultivation, callus tissue grew from the edges and intermediate cracks of the gradually browning, swelling, and cracking anthers. After 40 days, many callus tissues had grown to a size of 2–3 mm. The number of callus tissue blocks in each bottle of IM was counted, and the callus induction rate was calculated. The callus tissue was transferred to differentiation medium, and after 30–40 days of light cultivation, it differentiated into white or green seedlings. The numbers of white and green seedlings were counted, and the seedling rates were calculated. The callus induction rates (CIR) of IM4 and IM6 were the highest at 7.03% and 7.06%, respectively, with the lowest being only 3.09% for IM2 and an average of 4.73% for all IMs. The highest green seedling rate (GSR) was 3.23% for IM6, and the lowest was 1.67% for IM1 (Table 1), with an average of 2.40%. The highest white seedling rate (WSR) was 30.00% for IM5, and the lowest was 20.72% for IM4 (Table 1), with an average of 25.43%. Based on the comprehensive callus induction, green seedling, and white seedling rates, medium IM6 was considered to be the optimal IM for AC of HD9802S among all IMs. The differentiated green seedlings were transferred to root medium and planted according to the cluster lines. Thirty-three green seedlings were obtained, of which 10 were diploid, with a natural doubling rate of 30.30%.

### 2.3. Evaluation of Rice Blast Resistance

**Figure 2:**
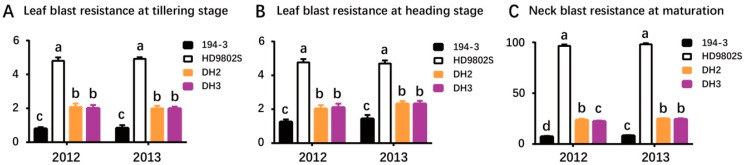
Phonotypes of improved lines and controls for rice blast under natural infection conditions Shown were the average values for leaf blast resistance at tillering (**A**) and heading (**B**) stages and neck blast resistance at maturation (**C**) of 194-3, HD9802S, DH2, and DH3 in 2012 and 2013. Phenotypic statistical data were expressed as mean ± standard deviation (SD), n = 24. Letters above the bars are ranked by the Duncan test at *p* < 0.05; different letters next to SD bars indicate a significant difference.

Molecular markers were identified in 10 doubled haploid (DH) lines obtained through AC using flanking markers L11-1 and I6. All plants were homozygous for these two markers, and seven of the 10 green seedlings, named DH1–DH7 according to their respective lines, contained *R6*. These results indicated that using MAS to screen donor materials for AC can reduce the blindness of material selection and increase the probability of obtaining green seedlings with the target gene in AC. After planting DH1–DH7 in a field in Wuhan, it was found that only DH2 and DH3 were pollen sterile, whereas the others were pollen fertile. The resistance of DH2 and DH3 to rice blast was identified in the naturally induced environment in Yuan’an (Table 2, Figure 2). Table 2 shows the comparative differences between the different lines and their parents. There were significant differences in the resistance levels to leaf blast, during tillering and heading, and neck blast between the two parents, 194-3 and HD9802S. The parent 194-3 showed high resistance to rice blast, whereas HD9802S was susceptible (Table 2, Figure 2). The resistance scores for leaf blast at the tillering and heading stages for the two DH lines were 1–2, indicating a high resistance level that was significantly higher than that of the control parent HD9802S (Table 2, Figure 2A,B). The incidence rate of neck blast in the two DH lines was 22–25%, and the resistance level of neck blast was level 2, whereas the incidence rate of HD9802S was close to 98%, which was level 9 (Table 2, Figure 2C). The results showed that the resistance of HD9802S to leaf blast at the tillering and heading stages and neck blast was greatly improved.

### 2.4. Characterization of Fertility

**Figure 3:**
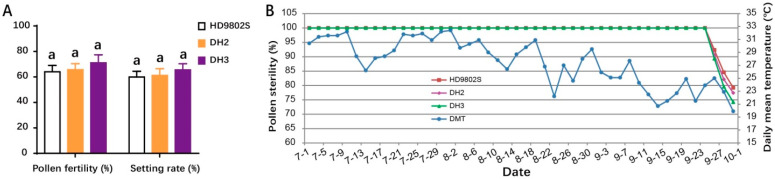
Fertility performance for the plants of HD9802S, DH2, and DH3 (**A**) Pollen fertility and bagging seed setting rates of HD9802S, DH2, and DH3 (n = 24). Letters above the bars are ranked by the Duncan test at *p* < 0.05; same letters next to SD bars indicate no significant difference. (**B**) Dynamic pollen fertility expressions of HD9802S, DH2, and DH3 relative to daily mean temperature (DMT) data from July 1 to September 30, 2012 (92 days in total) in Wuhan (n = 12).

HD9802S, DH2, and DH3 were planted in the experimental field at Huazhong Agricultural University in Wuhan in April, May, and June, respectively. At the fifth stage of young panicle differentiation (the phase from the formation of pollen mother cells), the average daily low-temperature treatment at 21 °C was carried out at the same time in a cold-irrigation tank to detect pollen fertility. Under natural growth conditions, the pollen from the three sowing periods (late June to late August) exhibited complete infertility. Random sampling and microscopic examination of pollen fertility revealed that the pollen in all three materials was completely sterile. After randomly selecting self-pollinated panicles with bagging, self-pollination sterility reached 100%, and agronomic traits were stable. After 1 week of low-temperature treatment at 21 °C in a cold-irrigation pool, the pollen fertility of HD9802S, DH2, and DH3 was 64.03%, 65.72%, and 71.11%, respectively, and the bagging seed setting rates were 59.95%, 61.18%, and 65.52%, respectively (Figure 3A). The fertility restoration of HD9802S, DH2, and DH3 can be used for breeding in a short-day, low-temperature growth environment during the winter in Hainan. The results showed that in the ecological environment during the normal sowing period in Wuhan, the fertility of DH2 and DH3 was stable, and pollen sterility remained at 100%, which was consistent with the pollen sterility of HD9802S. When subjected to low-temperature treatment at 21 °C for one week or more during the young panicle differentiation period, the fertility of HD9802S, DH2, and DH3 significantly fluctuated.

In China, a practical TGMS line needs to maintain a stable sterile period of more than 30 days in a local region. We observed the dynamic expression patterns of pollen fertility of HD9802S, DH2, and DH3 in the Wuhan experimental field at the end of summer 2012 and studied their stable sterile periods (Figure 3B). The results showed that DH2 and DH3 had a stable sterile period of 86 days, from 1 July to 25 September, during which they were completely male sterile, similar to HD9802S (Figure 3B). Analysis of pollen sterility data and temperature weather charts indicated that during the 5-day period from 12 to 16 September, the daily mean temperature (DMT) dropped below 23 °C (Figure 3B), which may affect the development of pollen grains of HD9802S, DH2, and DH3, resulting in partial fertility of their pollen on September 26. These results indicated that the sensitive period of temperature was 10 days before heading.

### 2.5. Investigation of Agronomic and Rice Grain Quality Traits

**Figure 4:**
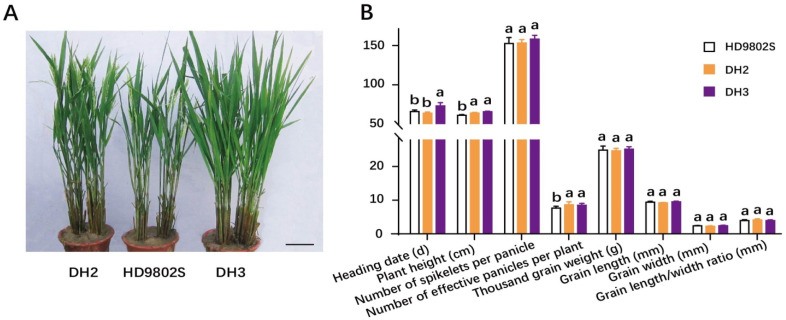
Performance of the main agronomic and rice grain quality traits of HD9802S, DH2, and DH3 Plant type. (**A**) and agronomic and rice grain quality traits Scale bar, 9.5 cm. (**B**) of HD9802S, DH2, and DH3. Phenotypic statistical data are expressed as mean ± SD, n = 24. Letters above the bars are ranked by the Duncan test at *p* < 0.05; different letters next to SD indicate a significant difference.

The improved lines DH2 and DH3 showed high rice-blast resistance and stable fertility. Meanwhile, we investigated important agronomic and rice grain quality traits, including plant height, heading date, effective panicles per plant, spikelets per panicle, 1000-grain weight, grain length, grain width, and grain length/width ratio for the two improved plant lines and HD9802S. The two improved lines and the control parent HD9802S were replanted three times in multiple residential areas in Wuhan. The results showed that there was no significant difference in heading date between DH2 and HD9802S, but the heading date of DH3 was significantly longer than that of HD9802S (Figure 4A,B). The plant height and effective panicles per plant of DH2 and DH3 were significantly higher than those of HD9802S (Figure 4A,B). Effective panicles per plant provided the potential for increasing yield. However, there were no significant differences in the number of spikelets per panicle, 1000-grain weight, grain length, grain width, and grain length/width ratio among HD9802S, DH2, and DH3 (Figure 4B). In summary, the two improved lines, DH2 and DH3, were different from their recurrent parent, HD9802S, in at least two agronomic traits, whereas the other agronomic traits were similar, and the phenotype of the recurrent parent was restored to a certain extent.

## 3. Discussion

### 3.1. Selection of AC Medium

AC is widely used in rice breeding, and techniques have been improved in many ways, including shortened breeding times, multiple genetic types, and high selection efficiency [24,25,26,27,29]. However, many technical and theoretical problems limit the widespread application of AC, such as low callus-induction, green seedling, and DH line improvement rates [24,25,38]. The callus induction and green seedling rates are related to the culture medium inoculated with anthers. In this study, we compared the effects of six IMs on callus induction and green seedling rates to select the optimal medium. According to previous studies on the IM for AC and the adjustment of the proportion of each component [25,31,32,38], the callus-induction rates of media IM4 and IM6 were 7.03% and 7.06%, respectively, with the highest green seedling rate of 3.23% (Table 1). This indicated that the *indica*-type TGMS line can induce callus formation and differentiation in seedlings. However, the callus induction and differentiation rates are both low. In the present study, it was found that adding 3 mg/L 2, 4-D, 2 mg/L NAA, and 1 mg/L KT to the MSIM significantly improved the callus-induction rate as well as the green and white seedling rates. This indicated that a combination of compound hormones can improve callus induction and green seedling differentiation rates. However, the addition of only NAA to the MS IM did not have a significant effect on callus induction, indicating that KT plays an important role and that there may also be some interaction between NAA and KT. Comparing the media IM2 and IM3, we found that adding 2% sucrose to the MS IM significantly reduced the white seedling rate and increased the callus-induction and green seedling rates (Table 1). Comparing the media IM5 and IM6, we found that the simultaneous addition of 800 mg/L CH, 600 mg/L Pro, and 500 mg/L Glu to the N6 IM significantly reduced the white seedling rate and improved the callus-induction and green seedling rates (Table 1). When using MAS before sampling, plants without target genes were excluded so that the improvement rate of the DH lines was increased. Ten DH lines were obtained, including seven lines containing *R6*, showing that the method was effective at obtaining DH lines containing *R6*. The combination of MAS, AC, and traditional breeding can quickly achieve breeding goals.

In this study, the callus induction and green seedling rate of six IMs were compared, and the proportion of different components was optimized. It was found that IM4 and IM6 were more suitable for the AC of HD9802S-background material. Only by continuously trying to find the most suitable culture medium for the target genotype can a large number of selectable regenerated seedling populations be obtained in a short period of time, which can be utilized for breeding. This study first used MAS of anther donors containing target genes, eliminated plants without target genes, reduced the sampling population, improved the selection efficiency of pollen green seedlings containing target genes, and achieved the goal of genetic improvement. Secondly, by comparing the effects of these six IMs on callus induction and green seedling rates, the optimal IM was selected, which is conducive to genetic improvement of other traits in the HD9802S background material through AC. Our findings demonstrate that the combination of MAS, AC, and traditional breeding can quickly achieve breeding goals.

### 3.2. Technology Roadmap for the Rapid Improvement of Target Phenotype by Combining MAS with AC

Two-line hybrid rice has been widely used in China to improve rice yields. However, many hybrids are highly susceptible to rice blast, which severely affects the spread of hybrid rice [11,13,19]. Therefore, it is important to improve the blast resistance of hybrid rice parents for the sustainable utilization of high-yield hybrids. With the increasing maturity of MAS technology, many studies have selected new high-quality and high-yield rice varieties that are resistant to rice blast and bacterial blight [11,13,19,20,21,22]. MAS combined with AC is one of the most economical and effective methods to rapidly address rice blast disease [31,32]. The selection of target genes is the first issue to be considered in MAS breeding. Extracting broad-spectrum high-resistance genes from germplasm resources with rich genetic diversity, especially wild rice germplasm resources, using MAS to select blast-resistance genes, and molecular design are powerful methods for coping with the rapid evolution of new biotypes [39]. At present, research on rice blast-resistance genes is mainly focused on a certain stage of rice growth, especially the seedling stage, but rice blast occurs in all growth stages. Previous studies have shown that *R6*, located on chromosome 6, is a blast-resistance gene throughout the entire growth period and a major gene for resistance to both leaf and neck blast identified in Yuan’an [35]. Currently, the genes that have been identified and cloned at similar positions include *Pi2*, *Pi9*, *Pi25*, *Pi26*, and *Pigm* [36]. Whether *R6* is an allele that has been cloned or mapped requires further investigation.

There are currently many successful studies on the genetic improvement of rice through BC_3_ [11,12,36]. Based on previous research, the present study obtained BC_3_F_1_ through one hybridization and three backcrosses, during which MAS and a gene chip were used to ultimately select materials with similar genetic backgrounds, agronomic traits, and high resistance to rice blast for AC. Among them, the two flanking molecular markers were heterozygous, as detected through insertion/deletion molecular makers. The population of BC_3_F_1_ was randomly detected using the gene chip, and the genetic background recovery of the recurrent parent in a single plant from BC_3_F_1_ was about 90.93%. This study obtained two DH lines, among which DH2 and HD9802S showed only significant differences in plant height, effective panicles per plant, and rice blast resistance. The plant height increased by about 2.83 cm, and the effective panicles per plant increased by about 1. Furthermore, the heading date of DH3 was significantly delayed by about 1 week compared to that of HD9802S. Compared to DH3, DH2 was closer to our goal, which was to improve the rice blast resistance of HD9802S without greatly changing the many agronomic traits. In this study, resistance to leaf blast at the tillering and heading stages and neck blast at the mature stage of lines DH2 and DH3 was significantly improved through the application of MAS (Table 2, Figure 2). This indicated that the use of MAS to introduce *R6* into HD9802S resulted in the selection of appropriate target genes and is feasible for improving rice blast resistance at various growth stages of HD9802S as well as improving the adaptability of HD9802S.

With the development of genome-wide high-throughput and high-resolution genotyping platforms, background selection based on genome-wide SNP arrays has become a feasible strategy for crop genetic improvement with high accuracy and efficiency [11,13,19]. To recover the maximum genetic background of the recurrent parent in a short period of time, breeders require a large backcross population and background selection based on a foreground check of the target gene [11,13,19]. The present study was based on MAS combined with the breeding chip RICE6K for the background detection of genetic improvements for rice blast resistance. After three backcrosses, all improved lines contained *R6* fragments from the donor (Figure 1). Two DH lines with stable fertility, DH2 and DH3, showed significant improvements in their resistance to rice blast. However, compared with the control HD9802S, there were certain differences in agronomic traits, such as plant height, heading date, and effective panicles per plant (Figure 4). The non-target genetic fragments from donor parent 194-3 affected the phenotypes of the DH lines. Further backcrossing and genetic background screening are beneficial for eliminating non-target fragments, resulting in an improved line phenotype closer to that of HD9802S. Simultaneous cloning of the *R6* gene and the development of functional molecular markers can also help reduce non-target fragments. Compared with HD9802S, DH2 had significantly increased resistance to rice blast (Figure 2), and at the same time, plant height increased to a certain extent (Figure 4). There was no significant difference in heading time, effective panicles per plant, spikelets per panicle, 1000-grain weight, grain length, grain width, and grain length/width ratio of DH2 (Figure 4), which was more of an improvement over HD9802S than DH3. Using a combination of MAS and AC alongside the identification of rice blast resistance in the field, investigation of agronomic traits, and screening of genetic background using breeding chips, the lines DH2 and DH3 with stable fertility and high blast resistance were obtained. This method quickly and effectively achieved our goals for genetic improvement and was demonstrated as an effective method for improving rice-blast resistance in the TGMS line.

## 4. Materials and Methods

### 4.1. Plant Materials and Field Planting

The parent 194-3 was provided by the Yichang Institute of Agricultural Sciences (Yichang City, Hubei Province, China). After years of identification in Yuan’an, Yichang, and Hubei Province, 194-3 showed long-lasting, high resistance to rice blast. HD9802S is a TGMS line bred by Hubei University (Wuhan City, Hubei Province, China) that is highly sensitive to rice blast. BC_3_F_1_ was constructed using HD9802S as the recurrent parent and 194-3 with *R6* as the male parent. Single plants with *R6* and favorable traits were selected as AC donor materials, from which both molecular markers were heterozygous. The phenotypic identification of rice blast in all materials was conducted in Yuan’an in 2011–2013. CO39, a variety that is highly susceptible to rice blast and was introduced by the International Rice Research Institute, was used to induce rice blast. Fertility, plant height, heading date, effective panicles per plant, spikelets per panicle, 1000-grain weight, grain length, grain width, and grain length/width ratio were investigated in Wuhan, Hubei Province, in 2012. During field data collection, the middle 10 plants in each row were selected.

All materials were sown on May 20 of the year, except for the material used for the dynamic observation of pollen fertility. Approximately 30 days after sowing, the rice was transplanted, and three replicates were set, in which two rows of 12 plants were planted in each line, with a row spacing of 16.50 cm × 26.40 cm. Field management was conducted according to local regulations.

### 4.2. Molecular Markers and Genotyping Used for MAS

Based on the results of fine mapping, two flanking molecular markers, L11-1 (F:5′-CAAGTTCTTAGGCTCTTAAT-3,’ R:5′-CAAGGATGATCAATGTCGAG-3′) and I6 (F:5′-GAAGAGCTGAGGTCGTCGGA-3,’ R:5′-AGACATAAGGTGAGAGGACA-3′), closely linked to *R6* were used to confirm the presence of *R6* for positive foreground selection. These two markers were developed based on the mapping of *R6* [35].

Total genomic DNA was extracted from the leaves of the plants using small CTAB samples. PCR was performed on a MyCycler™ thermal cycler (Bio-Rad, Foster City, CA, USA) using 20 μL system contained 2 μL DNA template (20 ng/μL), 1.4 μL dNTP (2 mM), 2 μL 10 × PCR Buffer, 0.2 μL each of the primer pair (10 μM), 0.1 μL Taq polymerase (10 U/μL) (all reagents purchased by Takara company, Tokyo, Japan), and used dH_2_O to water up to 20 μL. The PCR amplification reaction was run at 95 °C for 5 min prior to 33 cycles at 94 °C for 40 s, 55 °C for 30 s, 72 °C for 45 s, and a final extension at 72 °C for 7 min and 25 °C for 1 min before completion. After PCR amplification, 4% polyacrylamide gel electrophoresis was performed to directly detect the molecular marker genotypes of each plant.

The genetic background of the backcross material was identified using a RICE6K gene chip, which is a genome-wide SNP array based on Infinium technology [37]. Leaf DNA extraction, amplification, fragmentation, chip hybridization, single-base extension, staining, and scanning were performed by the Life Science and Technology Center, China National Seed Group Co., Ltd. (Wuhan, China).

### 4.3. Anther Culture

#### 4.3.1. Culture Medium

The anthers were placed on IM1 comprising MS medium, base salt, 3 mg/L 2,4-D, 800 mg/L CH, 600 mg/L Pro, 500 mg/L Glu, 30 g/L maltose, 20 g/L sucrose, and 3 g/L phytagel powder. This medium induces anthers to produce calli. The IM2–IM4 were supplemented with 2 mg/L NAA and 1 mg/L KT and were based on IM1, and the ratio of sucrose to maltose was adjusted at the same time (Table 1). IM5 and IM6 were N6 media, and IM6 contained 600 mg/L Pro and 500 mg/L Glu and was based on IM5. The differentiation medium was MS medium with 2 mg/L 6-benzylaminopurine, 2 mg/L KT, 0.2 mg/L indol-3-acetic acid, 0.2 mg/L NAA, 600 mg/L Pro, 1000 mg/L CH, 20 g/L maltose, 10 g/L sucrose, and 3 g/L phytagel powder. The rooting medium was 1/2 MS medium. All media had a pH of 6.0, and all reagents were purchased from Sigma, St. Louis MO, USA.

#### 4.3.2. AC Procedure

**Figure 5:**
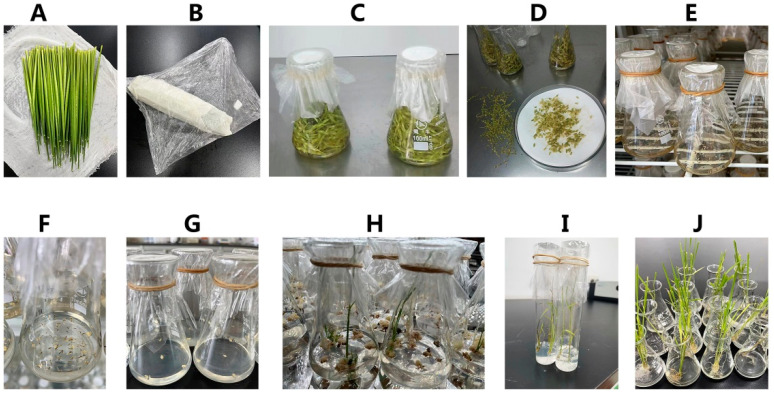
Steps of rice anther culture. (**A**) Rice young panicle collection and early treatment (**B**) Preparation of rice young panicle pre-treatment at low temperature (**C**) The screened young rice panicles were disinfected. (**D**) The screened rice spikelets were treated with hull cutting. (**E**) Rice anthers were inoculated on induction medium. (**F**) Rice anther-induced callus (**G**) The rice anther callus was differentiated. (**H**) The rice anther callus differentiated into green and white seedlings. (**I**) Green rice seedlings of anther culture (AC) for rooting (**J**) Green rice seedlings after AC were refined.

During the booting stage, the main panicle with a leaf pillow spacing of about 10 cm was used as the AC material in 2011. The leaves were cut off and the surface wiped with 70% alcohol until the alcohol was completely evaporated. The young ears were wrapped in wet gauze, sealed with plastic wrap, and stored at 8–10 °C for low-temperature pretreatment for 7–15 days. Young spikelets were stripped on an aseptic bench, sterilized with 0.1% HgCl_2_ for 10 min, and washed four times with sterile water. The spikelets were yellowish green, and their anthers were 1/2 to 1/3 the length of the spikelets and were cut into sterile Petri dishes. The anthers were inoculated into the IM by shaking the spikelets. Each 100 mL triangular flask was inoculated with about 50 anthers and cultured in the dark at 26 ± 2 °C. After 30–40 days of cultivation, the calli grew to 2–3 mm, and spherical, bright-yellow, dense-textured calli were selected and transferred to the differentiation medium. Callus tissues were differentiated under light (12 h of light per day) at 28 ± 2 °C. After about 40 d, the calli differentiated into AC plants. When the plants grew to about 5 cm, they were placed on the rooting medium for 2 weeks. After the green seedlings grew strong roots, they were cultivated for 1 week and transplanted into the field (Figure 5).

### 4.4. Rice Blast Evaluation

**Figure 6:**
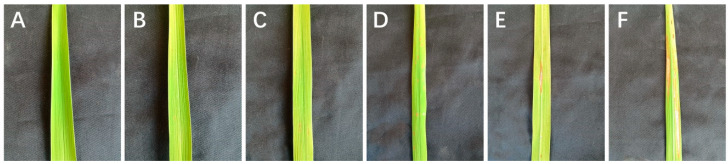
The 0–5-level scoring system of rice leaf blasts (**A**) Leaf without blast disease (**B**) Rice leaf blast of level 1. (**C**) Rice leaf blast of level 2. (**D**) Rice leaf blast of level 3. (**E**) Rice leaf blast of level 4. (**F**) Rice leaf blast of level 5.

The green plants cultivated in AC were numbered according to the differentiation source of calli and were planted in the blast resistance identification base in Yuan’an, which is an area that experiences a high incidence of rice blast [11,12]. The rice blast inspection method was based on previously reported standards [40]. Leaf blasts were graded using a 0–5 level scoring system, with levels 0–3 indicating resistance and levels 4 and 5 indicating susceptibility (Figure 6). The newly grown leaves of 10 individual plants in the middle of each line were examined by stage, and the most serious disease spots on the leaves were registered as leaf blast phenotypic values of this plant. Leaf blasts were investigated every 30 d during the tillering and heading stages, and neck blasts were evaluated 1 week before the maturity stage. The severity of the neck blast was recorded as the percentage of rice-spiked neck infections at physiological maturity. The number of infected plants showing the neck blast phenotype was expressed as a percentage of infection. Percentage resistance grading was used for neck blast, with rice blast rates from 0% to 25% indicating resistance and those from 26% to 100% indicating susceptibility.

### 4.5. Fertility Assessment

This study conducted fertility testing based on the general methods for pollen fertility identification and classified pollen morphology based on the IK-I staining of pollen grains; the pollen sterility of each spike was recorded. Lines with an average pollen sterility of over 99.5% were considered to be completely sterile [11,12]. The pollen staining rate was used as a fertility indicator. Anthers were selected from three to five spikelets in the middle and upper parts of a newly tilled panicle. The anthers were crushed on a glass slide, stained with a 1% I2-KI solution, and examined under a microscope (BK-FL4, Optec Instrument, Chongqing, China) in three different fields. The number of deeply stained pollen and non-stained or lightly stained pollen was counted, and the percentage of deeply stained pollen in the total pollen count was considered to be the pollen staining rate. Fertility testing was conducted on pollen treated at different sowing periods and at a low temperature of 21 °C in a cold-irrigation pond, with the pollen staining and bagging seed setting rates as indicators of fertility instability and conversion. For dynamic observations of pollen fertility under natural field conditions, we sowed TGMS lines every 15 days from 1 April to 1 July 2012, on the experimental farm of Huazhong Agricultural University (HAU). From 1 July to 1 October, dynamic studies with 2-day intervals were conducted under the microscope on the pollen fertility of each line of the first five spikelets of the primary panicle [12]. Pollen sterility data were recorded, with an average of 5 panicles per line. The Agricultural Meteorological Department of HAU recorded and provided DMT data.

### 4.6. Ploidy Identification

Referring to the method of Dolezel et al. [41], 1 g of fresh leaves was extracted and placed in 2 mL of cell lysate (0.18% Tris, 0.74% Na_2_EDTA, 0.01% Spermine, 5.8% KCl, 1.1% β-mercaptoethanol, 0.1% Triton X-100). Then the leaves were cut into pieces with a blade, filtered and collected filtrate, centrifuged at 5000 r/min for 5 min, and 2 mL of propidium iodide dye solution were added to the petri dish. After incubation in ice for 10 min in the dark, the nuclear DNA was fluorescently labeled, and the DNA content distribution map was automatically generated by the instrument. Using flow cytometry (MoFlo XDP, Beckman Coulter, Brea, CA, USA), diploid rice was used as the standard reference for each sample, and at least 20,000 particles were collected for each test. Each sample was repeated three times, and finally the average value was taken.

In the field, the distance from the top of the highest panicle to the ground after the seeds had matured into green seedlings was relatively low, and the glume was relatively short compared to that of the parents, making it difficult to bear fruit. This may be because the plants are haploid, which can be directly identified after planting in the field for a period of time [42]. The green seedling plants shortened, stems thickened, tiller number decreased, glumes thickened, grain size increased, and seed setting rate decreased. This may have been because of polyploidy [43].

### 4.7. Evaluation of Agronomic and Rice Grain Quality Traits

The distance from the top of the highest panicle to the ground after the seeds had matured was considered the plant’s height. The heading date of the plants was determined by the number of days from sowing to heading, using the main stem of the plant for heading. After maturity, 20 plants were sampled from the middle of each plot to determine the effective panicles per plant, spikelets per panicle, 1000-grain weight, grain length, grain width, and grain length/width ratio.

### 4.8. Data Statistical Analysis

The callus-induction, white seedling differentiation, and green seedling differentiation rates were calculated as follows: The ratio between the total number of induced callus tissues and the total number of inoculated anthers was the callus induction rate. The ratio between the total number of callus tissues in differentiated white seedlings and the total number of transferred callus tissues was the differentiation rate of white seedlings. The ratio of the total number of callus tissues in differentiated green seedlings to the total number of transferred callus tissues was the differentiation rate of green seedlings. Statistical testing was conducted using one-way analysis of variance (ANOVA) and Duncan multiple comparisons in IBM SPSS Statistics 22.0.

## 5. Conclusions

In this study, the *R6* gene was introduced into the rice-blast-susceptible cultivar HD9802S by backcross introduction and MAS. At the same time, materials containing *R6* suitable for AC were screened by combining gene chip, resistance identification, and agronomic traits in the field, and media suitable for HD9802S-background material were screened by six induction media. The two DH lines DH2 and DH3 with improved blast resistance were obtained at the same time. Compared with HD9802S, DH2 and HD3 increased plant height and the number of effective panicles per plant. In addition, HD3 increased the heading date. In short, we can quickly and effectively improve HD9802S through AC on the basis of combining genotype and phenotype identification to achieve our genetic improvement purpose.

## Figures and Tables

**Table 1:** Ratio of callus induction and green plant regeneration on four types of medium.

Medium	MS	N6	2,4-D mg/L	NAA mg/L	KT mg/L	CH mg/L	Pro mg/L	Glu mg/L	Surose g/L	Maltose g/L	No. of Anther	CIR (%)	No. of Plant	GSR (%)	WSR (%)
IM1	+	−	3.00	-	-	800	600	500	20	30	3000	4.00	2	1.67	29.17
IM2	+	−	3.00	2.00	-	800	600	500	-	30	4300	3.09	2	1.50	24.06
IM3	+	−	3.00	2.00	-	800	600	500	20	30	4600	4.00	5	2.72	21.74
IM4	+	−	3.00	2.00	1.00	800	600	500	20	30	5150	7.03	11	3.04	20.72
IM5	−	+	2.00	3.00	1.00	800	-	-	60	-	5600	3.21	4	2.22	30.00
IM6	−	+	2.00	3.00	1.00	800	600	500	60	-	3950	7.06	9	3.23	26.88

MS, Murashige and Skoog’s media; 2,4-D, 2,4-dichlorophenoxyacetic acid; NAA, 1-naphthaleneacetic acid; KT, kinetin; CH, casein hydrolysate; Pro, proline; Glu, glutamine; CIR, callus induction rates; GSR, green seedling rate; WSR, white seedling rate. No., number.

**Table 2:** Statistics of leaf blast resistance and neck blast resistance of 194-3, HD9802S, and two DH lines, DH2 and DH3.

Trait	Year	194-3	HD9802S	DH2	DH3
LT	2012	0.79 ± 0.10 ^c^	4.80 ± 0.21 ^a^	2.08 ± 0.20 ^b^	2.00 ± 0.20 ^b^
	2013	0.83 ± 0.18 ^c^	4.92 ± 0.10 ^a^	2.00 ± 0.15 ^b^	1.98 ± 0.12 ^b^
LH	2012	1.25 ± 0.15 ^c^	4.77 ± 0.19 ^a^	2.02 ± 0.22 ^b^	2.10 ± 0.23 ^b^
	2013	1.43 ± 0.23 ^c^	4.70 ± 0.18 ^a^	2.32 ± 0.16 ^b^	2.32 ± 0.18 ^b^
NB	2012	7.18 ± 0.80 ^d^	96.50 ± 1.38 ^a^	23.50 ± 1.38 ^b^	22.33 ± 0.82 ^c^
	2013	8.12 ± 0.66 ^c^	98.00 ± 1.55 ^a^	24.67 ± 0.82 ^b^	24.33 ± 1.03 ^b^

LT, leaf blast resistance at tillering stag; LH, leaf blast resistance at heading stage; NB, neck blast resistance at maturation stage. Phenotypic statistical data were expressed as mean ± SD, n = 24. Letters above the bars are ranked by the Duncan test at *p* < 0.05; different letters next to SD indicate a significant difference.

## Data Availability

For materials, please contact the corresponding author’s email address.
